# BRCA Mutations in Prostate Cancer: Assessment, Implications and Treatment Considerations

**DOI:** 10.3390/ijms222312628

**Published:** 2021-11-23

**Authors:** Sidrah Shah, Rachelle Rachmat, Synthia Enyioma, Aruni Ghose, Antonios Revythis, Stergios Boussios

**Affiliations:** 1 Department of Palliative Care, Guy’s and St Thomas’ Hospital, Great Maze Pond, London SE1 9RT, UK; sidrah.shah@nhs.net; 2 Department of Radiology, Guy’s and St Thomas’ Hospital, Great Maze Pond, London SE1 9RT, UK; rachelle.rachmat@nhs.net; 3 Department of Medical Oncology, Medway NHS Foundation Trust, Windmill Road, Gillingham ME7 5NY, UK; synthia.enyioma1@nhs.net (S.E.); antonios.revythis@nhs.net (A.R.); 4 Department of Medical Oncology, Barts Cancer Centre, St. Bartholomew’s Hospital, Barts Health NHS Trust, W Smithfield, London EC1A 7BE, UK; aruni.ghose@nhs.net; 5 Faculty of Life Sciences & Medicine, King’s College London, London WC2R 2LS, UK; 6 School of Cancer & Pharmaceutical Sciences, Faculty of Life Sciences & Medicine, King’s College London, London SE1 9RT, UK; 7 AELIA Organization, 9th Km Thessaloniki-Thermi, 57001 Thessaloniki, Greece

**Keywords:** prostate cancer, DNA damage repair, *BRCA* mutations, PARP inhibitors

## Abstract

Prostate cancer ranks fifth in cancer-related mortality in men worldwide. DNA damage is implicated in cancer and DNA damage response (DDR) pathways are in place against this to maintain genomic stability. Impaired DDR pathways play a role in prostate carcinogenesis and germline or somatic mutations in DDR genes have been found in both primary and metastatic prostate cancer. Among these, *BRCA* mutations have been found to be especially clinically relevant with a role for germline or somatic testing. Prostate cancer with DDR defects may be sensitive to poly(ADP-ribose) polymerase (PARP) inhibitors which target proteins in a process called PARylation. Initially they were used to target BRCA-mutated tumor cells in a process of synthetic lethality. However, recent studies have found potential for PARP inhibitors in a variety of other genetic settings. In this review, we explore the mechanisms of DNA repair, potential for genomic analysis of prostate cancer and therapeutics of PARP inhibitors along with their safety profile.

## 1. Introduction

Prostate cancer was the second most common cancer and ranked fifth in causing cancer-related death in men worldwide in 2020 [1]. Similar to other cancers, epigenetic and somatic or germline genetic modifications lead to higher risk of prostate cancer and its progression [2,3,4,5]. DNA damage is implicated in carcinogenesis and may occur spontaneously or secondary to endogenous sources such as reactive oxygen species and exogenous sources including ionising radiation, chemicals or toxins, and ultraviolet (UV) radiation [6,7,8]. DNA damage response (DDR) pathways are in place to maintain genomic stability by monitoring DNA integrity and activating the DNA repair process or induce cell apoptosis if necessary [9,10,11]. Impaired DDR pathways lead to genomic instability through survival and proliferation of unrepaired cells and subsequently tumorigenesis [12]. Among germline and somatic mutations in prostate cancer, DDR defects represent 25% of them—of these, *BRCA* mutations are the most frequent mutation to occur [13]. *BRCA1/2* genes are located at chromosome 17q21 and 13q12, respectively. They are large genes consisting of 100 and 70 kb, respectively. They have an autosomal dominant inheritance pattern with incomplete penetrance. There are multiple, major DDR pathways which are active during different phases of the cell cycle, and these include base excision repair (BER), nucleotide excision repair (NER), mismatch repair (MMR), homologous recombination (HR) and non-homologous end joining (NHEJ) [14]. In the case of impairment of HR, synthetic lethality induced by poly (ADP-ribose) polymerase (PARP) inhibition occurs and may target tumor tissue selectively. This review explores the DDR pathways with a particular focus on *BRCA* mutations, genomic analysis with testing guidelines and the role of PARP inhibitors in therapy for prostate cancer.

## 2. DNA Repair Pathways

### 2.1. Base Excision Repair (BER)

BER is critical for repair of small base lesions that do not distort the double DNA helix caused by oxidation, methylation and deamination [15,16]. In the nucleus, it is usually prominent in the G1 phase of the cell cycle [17]. BER is initiated by one of 11 DNA glycosylases to remove the damaged base lesion and create an abasic or apurinic/apyrimidinic (AP) site [14,18]. At this site, an AP-site specific AP endonuclease (APE1) incises the DNA backbone and either of two sub-pathways occur: the missing nucleotide is inserted by DNA polymerase β (POLβ) in a process called short-patch BER (the most dominant pathway usually), or 2–13 nucleotides are replaced by a variety of proteins in the long-patch repair pathway [19]. The replaced nucleotides are then sealed by a DNA ligase [18]. 

Kuasne et al. found mutations in BER repair gene APEX1 have been implicated as increasing prostate cancer risk (OR = 1.68 95% CI 1.10–2.58) [20]. Furthermore, the XRCC1 gene, responsible for bringing together DNA repair proteins such as DNA ligase 3 and DNA polymerase β, has also been linked to increasing prostate cancer risk [21].

### 2.2. Nucleotide Excision Repair (NER)

In contrast to BER, the NER pathway repairs bulky DNA adducts and lesions that may distort the DNA helix due to UV radiation, chemotherapeutic agents such as cisplatin and environmental mutagens such as benzo[a]pyrene [14,22]. There are two sub-pathways in NER called global-genome NER (GG-NER) and transcription-coupled NER (TC-NER) [14]. The GG-NER scans the entire genome including areas that are transcriptionally inactive, whereas in the TC-NER, RNA polymerase II will stall at the lesion on the transcribed active genes [23]. In GG-NER, recognition of DNA damage is primarily through a complex of xeroderma pigmentosum, complementation group C (XPC) protein, UV excision repair protein Radiation sensitive 23B (RAD23B) protein and Centrin 2 (CETN2), which detects single-stranded DNA [24,25]. Furthermore, the ultraviolet-damaged DNA damage-binding protein (UV–DDB) complex will bind to lesions secondary to UV damage and also allow binding from downstream repair proteins such as XPC [23]. Once there is recognition of damage by the XPC-RAD23B complex, it then binds to the 10-subunit transcription initiation factor II H (TFIIH) complex to open up the DNA, track for the lesion and then incise it [22]. DNA polymerases will then fill up the repair patch and be sealed by DNA ligases [23]. In the TC-NER pathway, RNA polymerase II will identify the lesion and recruit specific proteins Cockayne syndrome WD repeat protein A (CSA) and Cockayne syndrome protein B (CSB), which subsequently recruit other core proteins [26]. The complex translocates RNA polymerase II in reverse and therefore exposes the lesion; TFIIH will bind to the site and start the chain of events to remove the lesion as described for the GG-NER pathway [27]. 

Mandal et al. investigated the association between NER genes XPC PAT and XPC exon 15 and prostate cancer [28]. They found a significant association between XPC PAT Ins/Ins (I/I) genotype with a 2.5-fold risk of prostate cancer (Adjusted OR- 2.55, 95% CI-1.22–5.33, *p* = 0.012). Similarly, the XPC exon 15 variant CC genotype showed a 2.1-fold increased risk of prostate cancer (Adjusted OR- 2.15, 95% CI-1.09–4.23, *p* = 0.026). The Gleason grade categorises patients with prostate cancer based on cell differentiation; the XPC PAT I/I genotype displayed a 2.8-fold increased risk of a high Gleason grade (Adjusted OR-2.88, 95% CI 1.22–6.79, *p* = 0.015). These results highlight how variant mutations in NER genes can increase prostate cancer risk.

### 2.3. Mismatch Repair

Base mismatches and insertion-deletion loops (IDLs) that occur during replication are repaired by the MMR pathway [29,30]. There are eight genes which code for the MMR pathway: *hMSH2*, *hMSH3*, *hMSH5*, *hMSH6*, *hMLH1*, *hPMS1 (hMLH2)*, *hMLH3*, *hPMS2* (*hMLH4*) [31,32,33]. The protein of the *hMSH2* gene creates two separate heterodimers with *MSH6* or *MSH3*; these complexes MSH2-MSH6 (MutSα) and MSH2-MSH3 (MutSβ) identify and bind to the mismatches [34]. The MSH2-MSH6 complex identifies single base mismatches and up to two nucleotide IDLs, in contrast to the MSH2-MSH3 complex which identifies up to 13 nucleotide IDLs [35]. The MutSα or MutSβ recruits the MutL complexes—the most important of which is MutLα (MLH1/PMS2 heterodimer)—which act as a mediator for other proteins to remove the mismatch; these are proliferating cell nuclear antigen (PCNA), replication factor C (RFC) and exonuclease 1 (EXO1) [14,36,37]. In particular, the EXO1 protein removes the DNA towards and past the mismatch site; once excised, MutLα suppresses EXO1 activity [36]. Finally, a DNA polymerase will synthesise DNA to replace the excision and this will be sealed by DNA ligases [38].

MMR deficiency has a prevalence of 3–12% in prostate cancer [39]. Graham et al. identified 27 men with MMR deficiency metastatic prostate cancer—the MSH2 gene was the most frequently mutated (20 men, 74%). 13 men (48%) had M1 metastatic disease on diagnosis and 19 out of 24 men (79%) who had a prostate biopsy had a Gleason score ≥ 8. They highlighted that MMR deficiency is associated with a high Gleason score and advanced metastatic disease on diagnosis, but further studies with a higher sample size are needed to investigate this further [39].

### 2.4. Homologous Recombination and Non-Homologous End Joining

**Figure 1:**
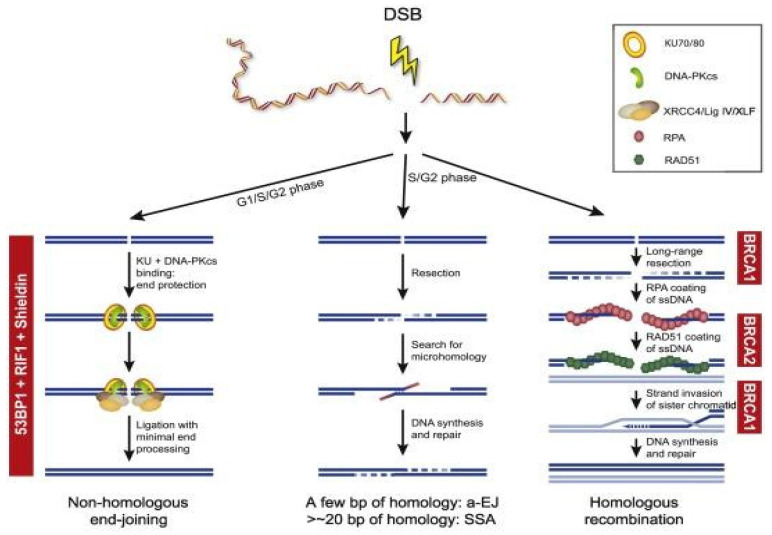
Overview of the different DSB repair pathways. End resection is the essential step for the choice between cNHEJ and HR, a-EJ, and SSA. The availability of a sister chromatid will direct HR. Different steps during HR are dependent on BRCA1 and BRCA2. NHEJ is dependent on the activity of 53BP1, RIF1, and the Shieldin complex (REV7, SHLD1, SHLD2, and SHLD3).

DNA double-strand breaks (DSBs) are repaired predominantly through HR and NHEJ [40]. HR occurs in the S and G2 phases of the cell cycle as it requires a template of a sister chromatid and will repair the DNA damage error-free; compared to NHEJ which occurs throughout the cell cycle, but especially in the G1 phase and is error-prone as it ligates the ends of broken DNA without a template [4,41]. In NHEJ, both free ends of DSBs will be recognized and bound by the Ku70/80 heterodimer and this recruits the DNA-PK catalytic subunit (PKcs) to form a multi-unit complex [42]. This complex then recruits further proteins such as Artemis and PNK to repair into a normal DNA structure, polymerases to bind the broken ends, and ligases to seal both DNA strands [43,44,45,46]. The NHEJ process is error-prone without using a DNA template and can consequently drive genomic instability [4]. In contrast, HR is predominantly error-free due to identical sister chromatids being present [47]. Damaged DNA will be identified and the MRN complex (MRE11-NBS1-RAD50) will be recruited, which will activate ATM and the RAD3-related ATR kinase [48]. This will stop the cell cycle to allow DNA repair to occur and create 3′-single-stranded DNA (ssDNA) ends [47]. The exposed ssDNA will be coated by Replication Protein A (RPA), which will be replaced by *Rad51* in a BRCA1/2 dependent pathway to complete the recombinase reaction [49]. The *BRCA1/2* genes are key mediators in the HR pathway and any *BRCA* mutations would impair this HR pathway [4,50]. For example, *BRCA1/2* mutations can lead to HR deficiency which would allow tumorigenesis [51]. Figure 1 provides an overview of the different DSB repair pathways.

Germline *BRCA* mutations have been linked to reduced metastasis-free survival in localized prostate cancer and the importance of detecting these mutations will be discussed below [52].

### 2.5. Synthetic Lethality

Synthetic lethality is a phenomenon where inactivation of one gene allele allows cell survival compared to cell death when multiple gene alleles are disrupted [53]. Cancer cells suffer from increased oxidative stress levels and subsequent DNA damage [50,54]. DDR pathways would usually repair this DNA damage, but DDR inhibitors have been used to achieve synthetic lethality that leads to tumor cell apoptosis [50]. An example of this are the PARP inhibitors which have been used in treatment of prostate cancer [55]. PARP enzymes repair single-strand breaks and can also be activated in the HR repair (HRR) pathway for DSBs [53]. Therefore, PARP inhibitors lead to accumulation of single-strand DNA breaks along with DSBs, which would physiologically be repaired through the HRR pathway with BRCA1/2 and PARP [53]. However, in *BRCA* mutated prostate cancer, PARP inhibition would prevent tumor DNA repair and lead to tumor cell apoptosis [53]. Therefore, inactivation of both demonstrates synthetic lethality where tumor cells can no longer survive. Synthetic lethality could also be used in tumors which share molecular features of *BRCA* mutated tumors—known as “BRCAness” [56]. Therefore, mutation of genes other than *BRCA* in the HR pathway allows for greater therapeutic use of PARP inhibitors and there is ongoing research into this broader use of synthetic lethality targeting the HR pathway [57]. 

In summary, there are multiple DDR pathways which protect genome stability. Mutations can occur in these DDR genes and increase susceptibility to prostate cancer. The most common mutation is of the *BRCA* genes, which are a vital part of the HR pathway and can lead to more aggressive disease. Therefore, there has been a focus on BRCA-mutated prostate cancer in the research. Treatment for BRCA-mutated prostate cancer includes PARP inhibitors, which use the phenomenon of synthetic lethality to increase single-strand breaks and DSBs of tumor DNA—which cannot be repaired through a deficient HR or PARP inhibition—and therefore cancer cell death [58]. Their use has also expanded to target prostate cancer with non-BRCA mutated genes, but have similarity to the BRCA mutated genes [57].

## 3. Genomic Analysis of Prostate Cancer

Prostate cancer has a high inheritable component and genomic analysis allows identification of a mutation in screening high-risk individuals along with driving treatment decisions with specific therapeutic agents [59]. The Cancer Genome Atlas (TCGA) found molecular heterogeneity after analysis of 333 primary prostate cancer specimens [60]. Inactivation of DDR genes were found in 19% of localized prostate cancers with the majority in the HR pathway—these included *BRCA1* (1%), *BRCA2* (3%), *RAD51C* (3%). Furthermore, other vital kinases involved in DNA repair were inactivated: *CDK12* (2%), *ATM* (4%), *FANCD2* (7%) [60]. Other studies have compared the mutations found in primary and metastatic prostate cancer. Pritchard et al. analyzed germline mutations of 20 DNA repair genes in 692 men with metastatic castration-resistant prostate cancer (mCRPC) and no family history; 84 deleterious mutations in these DNA repair genes were identified in 82 men (11.8%) with the most frequent being *BRCA2* at 5.3% [61]. In comparison, the Cancer Genome Atlas prostate cancer study found 4.6% of men with localized prostate cancer had germline mutations in these 20 DNA repair genes [16,61].

Grasso et al. also compared 50 mCRPC with 11 high-grade localized prostate cancer [62]. They found higher mutations in DNA repair factors such as *BRCA2*, *ATM* and *RAD50* in mCRPC (46%) compared to localized prostate cancer (27%) [61]. Furthermore, the study by the International Stand Up to Cancer/Prostate Cancer Foundation team (SU2C-PCF) analyzed 150 metastatic prostate cancer specimens and identified 8% with germline DDR mutations and 23% with somatic DDR alterations [63]. Of the samples, *BRCA2* was the most frequently mutated (13%), followed by *ATM* (7.3%), *MSH2* (2%) and *BRCA1*, *FANCA*, *MLH1*, *RAD51B* and *RAD51C* (0.3% for all) [61]. Germline *BRCA2* mutations were identified in 5.3%, which is higher than observed in primary prostate cancer [16,61]. PROREPAIR-B was the first prospective study to determine the prognostic impact of *BRCA2* as well as *BRCA1*, *ATM* and *PALB2* on cause-specific survival (CSS) in 419 mCRPC patients [64]. The study did not reach the primary end-point as there was no statistically significant difference in CCS between non-carriers and carriers of *ATM*, *BRCA1/2* and *PALB2* (33.2 months vs. 23.3 months, *p* = 0.264). However, germline *BRCA2* mutation was identified to be a negative prognostic factor on CSS at 17.4 months compared to 33.2 months in non-carriers (*p* = 0.027). Therefore, BRCA2 mutation was confirmed as an independent prognostic factor for CSS (*p* = 0.033) [64]. 

These studies demonstrate both germline and somatic mutations, especially in DDR genes, are prevalent in both primary and metastatic prostate cancer. Testing for these mutations early on could identify those who may progress to advanced metastatic disease as well as specific individualized treatment options [65]. Germline mutation testing allows screening in men who are of risk of prostate cancer, while somatic mutation testing can determine treatment options [66]. 

The advent of PARP inhibitors in mCRPC has popularized germline testing of mutations in DNA repair genes such as *BRCA1* and *BRCA2* [67]. Guidelines for the above have been framed as a necessity in an era of precision oncology. The National Comprehensive Cancer Network (NCCN) Prostate Cancer Guideline Version 4.2019 recommends germline testing for *BRCA1*, *BRCA2*, *PALB2*, *ATM*, *CHEK2*, *FANCA*, *RAD51D* and *HOXB13*. Criteria include risk group, pathology and family history. All metastatic PC under the high or very high risk NCCN groups warrant testing. Low to intermediate risk groups are tested in the presence of intraductal pathology or a background of suspicious family history. This implies males in the family diagnosed with PC less than 60 years of age or those who died from it, Ashkenazi Jewish ancestry, or having at least three cancers running in the family consistent with hereditary breast ovarian cancer (HOBC) or Lynch syndrome [68]. The NCCN Genetic/Familial High-Risk Assessment: Breast, Ovarian, and Pancreatic Guideline (Version 1.2020) recommend testing specifically for high penetrance genes including BRCA1 and BRCA2. Criteria include men with: (1) metastatic prostate cancer or intraductal pathology; (2) Gleason ≥7 of Ashkenazi Jewish ancestry; (3) ≥1 first, second or third degree with breast cancer diagnosed ≤50 years of age or ovarian/pancreatic/metastatic prostate cancer/intraductal prostate cancer diagnosed at any age; (4) ≥2 first, second or third degree relative with breast cancer or prostate cancer irrespective of Gleason and age of diagnosis [69].

The Philadelphia Prostate Cancer Consensus Conference 2019 was an international effort to gain a multi-disciplinary consensus among oncology, urology, cancer genetics, epidemiology, patient advocates, and NCCN leaders to arrive at uniform guidelines for germline testing. It was recommended for men with mCRPC and castration-sensitive prostate cancer and for men with strong family history of two or more male relatives diagnosed with prostate cancer at <60 years of age or died from the same. Additional considerations for testing included pathologic criteria (intraductal pathology; advanced disease of T3a or higher; or International Society of Urological Pathology [ISUP] Grade Group 4 or above) and family history criteria (two or more cancers in the HBOC or Lynch syndrome spectrum diagnosed at <50 years of age or having Ashkenazi Jewish ancestry) [70]. The NCCN Prostate Cancer Early Detection Guideline Version 2.2019 recommends considering *BRCA1/2* status for prostate cancer screening starting at 40 years of age or to consider annual screening vs every two-year screening among *BRCA1/2* mutation carriers [68]. The NCCN Genetic/Familial High-Risk Assessment: Breast, Ovarian, and Pancreatic Guideline Version 1.2020 recommends Prostate Specific Antigen (PSA) screening start at 40 years of age for *BRCA1* and *BRCA2* carriers [69]. The 2019 Philadelphia Prostate Cancer Consensus Conference recommended that *BRCA2* mutation status be a part of prostate cancer screening discussions and to start prostate cancer screening at age 40 or 10 years prior to the youngest prostate cancer diagnosed in a first-, second- or third-degree male relative [70]. 

The European Society for Medical Oncology (ESMO) Clinical Practice Guidelines for prostate cancer recommend tumor testing for homologous recombination genes and mismatch repair defects (or microsatellite instability) in all patients with mCRPC. Patients with pathogenic mutations in cancer-risk genes identified through tumor testing should be referred for germline testing and genetic counselling. Germline testing for *BRCA2* and other DDR genes associated with cancer predisposition syndromes is warranted in patients with a family history of cancer [71]. The European Association of Urology-European Association of Nuclear Medicine-European Society for Radiotherapy and Oncology-European Association of Urology Section of Urological Research-International Society of Geriatric Oncology (EAU-EANM-ESTRO-ESUR-ISUP-SIOG) guidelines also recommends germline testing in metastatic prostate cancer. Their criteria include—men with (1) high-risk prostate cancer with a relative diagnosed with prostate cancer at age < 60 years; (2) multiple relatives diagnosed with prostate cancer at age < 60 years or died from it; (3) family history of high-risk germline mutations or multiple cancers on same side of family. However, the strength of these recommendations is “weak” [72]. 

Contrary to the above advocacy of germline testing all around the globe, the United Kingdom’s National Institute for Health and Care Excellence (NICE) does not recommend the PARP inhibitor olaparib for the treatment of patients with hormone-relapsed, metastatic prostate cancer that harbour *BRCA1* or *BRCA2* mutations that have progressed on abiraterone or enzalutamide. According to them, treatment options for the above cohort include docetaxel, cabazitaxel or radium-223. Although clinical trial data have demonstrated that patients who received olaparib have extended progression-free survival (PFS) and overall survival (OS) over those who received re-treatment with either abiraterone or enzalutamide, evidence is unclear because re-treatment with those agents is not considered to be effective and is not standard of care in the National Health Service (NHS). It is also uncertain how effective olaparib is compared with cabazitaxel, radium-223 or docetaxel because there is no evidence directly comparing them. Hence, NICE is one of the very few which have no guidance on germline testing of prostate cancer [73]. 

## 4. DDR Mutations in Prostate Cancer 

### 4.1. Germline Mutations in DDR Genes 

Prostate cancer has a high rate of genomic instability, including amplifications, deletions, and chromosomal rearrangement [74]. This is usually a result of DNA damage; therefore the role of DNA repair genes is crucial. As mentioned previously, germline mutations in DDR genes have been identified with a particular focus on the *BRCA1/2* [75]. However, Nicolosi et al. studied these and other germline mutations in 3607 men with a history of prostate cancer between 2013 and 2018 [75]. They found 620 men (17.2%) had germline mutations with only 30.7% of these being *BRCA1/2* mutations; other mutations included *ATM*, *PALB2*, *CHEK2* and mismatch repair genes *PMS2*, *MLH1/2/6*. Their findings of mutations are summarized and compared to the Pritchard et al. study of metastatic prostate cancer previously discussed in Table 1. *BRCA1* and *BRCA2* have been identified as increasingly frequent in advanced metastatic disease compared to primary tumors [61]. In a study of 2000 patients with localized prostate cancer, including *BRCA1* and *BRCA2* mutation carriers, 23% of the mutation carriers developed metastatic disease five years after radical treatment [76]. This was increased compared to noncarriers at 7%. The cause specific survival was also reduced in *BRCA1/2* mutation carriers (8.6 years to 15.7 years in noncarriers). A subgroup analysis concluded that *BRCA2* mutations are an independent factor for poor prognosis.

ATM and ATR are signalling kinases activated by DSBs and replication stress, respectively, which can activate other signalling proteins such as CHEK2; inactivation of these genes causes androgen-induced instability and carcinogenesis [77,78]. A case study of men with prostate cancer with ductal histology, showed that 49% had DDR gene alterations; in particular, 20% were germline alterations, 14% were somatic gene mutations (mismatch repair gene alterations) and 31% carried mutations in homologous recombination repair genes [79]. Isaacsson Velho et al. have suggested that germline DNA repair mutations are more prevalent in recurrent or metastatic prostate cancer, ductal/intraductal histology and lymphovascular invasion [80].

### 4.2. Somatic Mutations in DDR Genes

In addition to germline mutations, somatic mutations also drive prostate carcinogenesis especially into the advanced stages [2]. Oncogenes or inactivation of tumor suppressor genes (TSGs) can lead to uncontrolled tumor growth. The TSG *p53* is commonly mutated in prostate cancer; 10–20% of primary and up to 42% of advanced prostate cancer [81,82,83]. Similarly, the TSG *PTEN* mutations are found in 27% of primary and up to 60% of metastatic prostate cancer [84,85,86]. The *AR* is a specific oncogene especially implicated in prostate cancer as AR-directed transcription (androgen dependent/independent) allows growth of the tumor in all stages of prostate cancer [75,87]. These somatic mutations are present in DNA repair pathways—Robinson et al. found 23% of mCRPC had somatic DDR mutations [63]. *BRCA1/2* and *ATM* accounted for nearly 20% of these and were found more frequently in mCRPC. Furthermore, loss of *BRCA2* was found in 12.7% of cases and 90% displayed biallelic loss [55]. Abida et al. also found that somatic *BRCA2* mutations were exhibited in tumors early on that then progressed to metastatic disease [88].

### 4.3. BRCA Genes-Functional Similarities and Differences between BRCA1 and BRCA2

Breast cancer predisposing genes *BRCA1* and *BRCA2* genes hold a crucial role as DNA repair genes and regulators of transcription, and are also implicated in ovarian, prostate, and pancreatic cancer [89,90,91,92,93,94]. Among the germline mutations, *BRCA1* and *BRCA2* tumor suppression genes are inherited in an autosomal dominant manner with incomplete penetrance [95]. For tumorigenesis to occur, the wild-type allele needs to be inactivated [90]. *BRCA1* gene encodes large proteins that are involved in cellular control systems, such as in DNA damage response and repair, chromatin modeling and transcriptional regulation [90].

*BRCA1* coregulates the androgen receptor (AR) which mediates a signalling pathway crucial in developing prostate cancer [96,97]. However, regulation of IGF-1 in an AR dependent manner has also been described as an action of the *BRCA1* gene [98]. Additionally, Bednarz et al. found that in a cohort of sporadic prostate cancer patients treated with radical prostatectomy, there was a higher probability of advanced tumor stage and a reduced disease-free survival associated with somatic *BRCA1* loss [99]. The somatic *BRCA1* loss was due to hypermethylation or a deletion of the promoter. Other mechanisms of BRCA1in cancer include promotion of loose-end resection and aiding RAD51 loading onto DNA, competing with p53-binding protein 1 (53BP1) in binding at DSBs and assists in determining whether repair is shunted toward HR or NHEJ [100,101]. In the *BRCA2* gene, a prostate cancer cluster region (PCCR) has been identified, in which the oligonucleotide/oligosaccharide- binding domain 1 (OB1) and Tower domain 2 (OB2) correlated with the highest risk of prostate cancer [102]. In a multinational study of 6500 male patients with *BRCA1* and *BRCA2* pathogenic sequence variants (PSV), two regions in BRCA2 (c.756-c 1000 and c.7914þ) were identified as high risk for developing Gleason 8b prostate cancer—this was more prominent in PSVs in PCCRs [103]. *BRCA2* function is limited to DNA recombination and repair processes, especially in regulation of RAD51 activity [90,104]. 

Functional loss of *BRCA1* and *BRCA2* is associated with deficiency in repairing DSBs through HR [105]. In their absence, DNA repair is happening by nonconservative and potentially mutagenic mechanisms. This genomic instability is thought to be responsible for the cancer predisposition caused by deleterious mutations in *BRCA* genes [100]. However, the correlation with specific sites, such as breast, ovarian and prostate cancer is currently still being researched. Francis et al. suggested that the *BRCA2* gene may act as a tumor suppressor gene in epithelial prostate tissue and when not functionally active, predisposes to prostate premalignant lesions [106]. In their study, deletion of *BRCA2* in murine prostatic epithelia led to focal hyperplasia and low-grade prostate epithelial neoplasia (PIN) in animals aged over 12 months. In simultaneous deletion of *BRCA2* and *TP53*, focal hyperplasia and atypical cells were observed at six months and prostatic intraepithelial neoplasia at 12 months of age [101]. Additionally, Moro et al. found a functional *BRCA2* gene was suggested to limit the metastatic potential of neoplastic cells by inhibition of PI13 Kinase/ATK and activation of MAP/EPK, and therefore downregulating matrix metalloproteinase 9 (MMPO9) [107]. This is thought to result in protection from cell migration and invasion.

### 4.4. Inherited Mutations in DDR Genes and Prostate Cancer Risk

Germline testing in men with prostate cancer can guide the relevant treatment options and screening of other linked cancers or family members if a mutation is identified [108]. Relatives with germline *BRCA1* and *BRCA2* mutations could be at increased risk of male breast, colon, pancreatic and prostate cancer [103]. For example, in a study of 913 male carriers of germline *BRCA1* mutation, the relative risk of prostate cancer was increased by 3.75-fold with an 8.6% cumulative risk by the age of 65 [109]. The IMPACT study (Identification of Men with a Genetic Predisposition to Prostate Cancer: Targeted screening in germline *BRCA1/2* mutation carriers and controls) facilitated annual PSA screening in families with germline BRCA1/2 mutations [110,111]. A total of 2,932 men with no history of prostate cancer were categorized into 919 germline BRCA1 carriers, 902 germline *BRCA2* carriers, 479 germline *BRCA2* non-carriers and 709 germline *BRCA1* non-carriers. Of these, 199 men (8.0%) had a PSA of >3.0ng/mL and were referred for a prostate biopsy—162 of these men (81.4%) subsequently had biopsy. 59 of the completed biopsies (36.4%) showed prostate cancer. The positive predictive value (PPV) of the prostate biopsy—number of cancers found divided by the number of biopsies—when PSA was >3.0ng/ml was 48% in *BRCA2* carriers, 33.3% in *BRCA2* non-carriers, 37.5% in BRCA1 carriers and 23.3% in *BRCA1* non-carriers. There was no significant difference observed between *BRCA1* carriers and non-carriers. While not statistically significant, there was a higher PPV in *BRCA1* and *BRCA2* carriers. However, with the highest in *BRCA2* carriers, the study suggested that PSA testing in this population with a threshold of 3ng/ml for biopsy may have increased specificity and detect early-stage disease, therefore reducing morbidity and mortality [111].

## 5. Implications for the Treatment

The first line treatment of metastatic prostate cancer is androgen deprivation therapy (ADT), which can be combined with chemotherapy agents; yet the majority eventually develop resistant disease defined as castration resistant prostate cancer [112]. Thus, new clinical targets are essential [41,113]. This section will discuss PARP inhibitors in detail along with their conducted clinical trials. PARP inhibitors alter, by PARylation, target proteins with ADP-ribose, a process present in several cellular processes such as cell growth and differentiation, transcriptional regulation, and apoptosis [65]. PARP has a significant role in DNA repair during complex signalling pathways in DNA damage response [41,114]. PARP is involved in single strand break repair, hence why they are particularly effective as therapeutics in this pathology [41,65]. The mechanism of action of PARP inhibitors have led to the current use of PARP inhibitors in cancer treatment, particularly targeting BRCA-mutant cancer cells and, more recent studies have shown a wider therapeutic potential beyond BRCA-mutant cancer cells [41,115]. Multiple clinical trials are studying PARP inhibitors as either monotherapy or part of combination therapy for prostate cancer. 

### 5.1. Clinical Development of PARP Inhibitors in Prostate Cancer

In the last decade, translational and multiple basic laboratory studies have researched functions of PARP1. Prostate cancer comprises multiple genetic events, for example, rearrangements of androgen responsive genes—commonly TMPRSS2—and ETS transcription factors, which PARP can target [116,117]. 

Furthermore, *BRCA1* and *BRCA2* mutant cells are sensitive to PARP inhibitors, and in vivo and ex vivo studies have shown PARP inhibitors to reduce AR function, resulting in reduced castration resistance prostate cancer growth [65,111,118]. Studies have shown that PARP inhibitors may block prostate cancer disease progression to castration resistance prostate cancer, with reduced castration resistance growth and delayed development from local disease to mCRPC [111]. Furthermore, PARP inhibitors may also be effective in prostate cancer with genes indirectly involved in the HR pathway that are similar to BRCA mutations [119]. Several PARP inhibitors have been developed and investigated in clinical research, six of which are summarized in Table 2.

### 5.2. PARP Inhibitors and Synthetic Lethality 

#### 5.2.1. TOPARP-A and TOPARP-B Studies

The Trial of PARP Inhibitor Prostate Cancer (TOPARP) studies evaluated the effectiveness of a PARP inhibitor, olaparib, and identified predictive markers and molecular pattern of prostate cancer cells [54,67,120]. 

The TOPARP-A phase II was an open-label, single-arm, and two-part adaptive trial on the PARP inhibitor olaparib [67]. The trial recruited 50 (30 in stage one and 20 in stage two of the trial) mCRPC patients who had progressed through one or two lines of chemotherapy. In the first part of the trial, olaparib 400 mg twice daily was given to genetically unselected participants until unequivocal disease progression, unacceptable toxicities, withdrawal of consent or death [67]. The primary end point was the objective response rate (ORR) outlined in the modified Response Evaluation Criteria in Solid tumors (RECIST); a greater than 50% reduction in PSA levels, a reduction in circulating tumor cells count (CTC) from five or more (at baseline) to less than five cells per 7.5 mL of blood [65,67,125]. The trial met end point on a basis of composite response with 33% response rate (95% confidence interval [CI] 20–48), 22% reduction in PSA level of 50% or more and 29% reduction in CTC. Although the trial showed a high response rate in biomarker positive patients, there were some biomarker positive tumors which did not respond to the treatment. Olaparib was overall well tolerated by the participants with a median treatment duration of 12 weeks. 

In the second part of the study, pre-planned biomarker analysis was performed and 33% of patients had homozygous mutations, deletions, or both in DDR genes including *ATM*, Fanconi’s anaemia genes (*FANCA*), *BRCA1/2*, *HDAC2*, *CHEK2*, and *PALB2*. 88% of these patients had a response to olaparib, including 80% with *ATM* (but no radiological response) and all patients with *BRCA2*. On the other hand, only two of 33 biomarker-negative patients (6%) had a response to olaparib (sensitivity, 88%; specificity, 94%). These results reinforced the notion of synthetic lethality of PARP inhibitors in impaired DSBs repair along with the TOPARP-B trial [67,120,126]. TOPARP-B was a phase II multicentre, open-label, randomized trial which included 92 pre-treated mCRPC patients, randomized to a 1:1 ratio to receive olaparib 400 mg or 300 mg twice daily [120]. Again, the primary end point was defined by RECIST criteria as in TOPARP-A. The trial showed a composite response achieved in 54% (95% Cl 39.0–69.1) of patients receiving 400 mg twice daily and 39% (CI 25.1–54.6) of patients receiving 300 mg twice daily. Although the composite response in patients treated with 300 mg twice daily was lower, 30% of patients on the higher dose of olaparib developed grade 3–4 adverse events, leading to dose reduction or discontinuation of treatment. This trial showed patients with germline or somatic *BRCA2* mutations had the best response to olaparib compared to patients with *ATM* or *CDK12* mutations.

#### 5.2.2. PROFOUND Study

The PROFOUND phase III randomized, open-label trial included 387 mCRPC patients with disease progression despite AR signalling inhibitor treatment [121]. Patients were divided into two cohorts with cohort A (245 patients) containing patients with BRCA1/2 or ATM mutations and cohort B (142 patients) with patients having all other DDR gene mutations. Participants were randomly assigned in a 2:1 ratio to have either olaparib 300 mg twice daily or prednisolone and an AR signalling inhibitor (enzalutamide or abiraterone) as part of the control group. The primary end point was radiological PFS (rPFS). Patients in cohort A had a significantly higher rPFS, with a median of 7.4 months compared to 3.6 months for patients in the control group (hazard ratio for progression or death: 0.34; 95% CI 0.25 to 0.47; *p* < 0.001) [121]. Median OS in cohort A was 18.5 months in the olaparib group compared to 15.1 months in the control group (hazard ratio for death, 0.64; 95% CI, 0.43 to 0.97; *p* = 0.02), and 81% of patients with disease progression crossed over to receive olaparib at the investigators’ discretion. 

In both cohorts A and B, the rPFS was significantly longer in those receiving olaparib compared to the control group (5.8 months vs. 3.5 months; hazard ratio, 0.49; 95% CI, 0.38 to 0.63; *p* < 0.001). The confirmed ORR (defined as the percentage of patients who had complete or partial response based on imaging) was 22% in patients treated with olaparib and 4% in the control group (odds ratio, 5.93; 95% CI, 2.01 to 25.40). At interim analysis, the median OS was 17.5 months in patients receiving olaparib and 14.3 months in patients in the control group (hazard ratio for death, 0.67; 95% CI, 0.49 to 0.93). Patients with *BRCA1/2* mutations had better therapeutic response to olaparib compared to patients with *ATM* or *CKD12* mutations, similar to results noted in TOPARP-B trial. Based on the positive preliminary results, the Food and Drug Administration (FDA) in May 2020 approved olaparib in mCRPC patients with deleterious HR gene mutations with disease progression following AR signalling inhibitor treatment [65]. 

#### 5.2.3. TRITON2 and GALAHAD Studies

The TRITON2 and GALAHAD phase II trials investigated therapeutic benefit of rucaparib and niraparib, respectively, two other PARP inhibitors, in mCRPC patients who had DDR mutations and disease progression despite AR signalling inhibitor or taxane-based chemotherapy [122,123,124]. In the TRITON 2 trial, 190 patients with mCRPC were treated with rucaparib 600 mg twice daily [122]. 52% of the patients had a *BRCA1/2* mutation and the remaining had *ATM* (30%), *CDK12* (7%), *CHEK2* (4%) and other mutated genes (7%). The ORR was 44% for patients with *BRCA* genes, but 9.5% for ATM and 0% for CDK12, *CHEK2* and the other DDR genes [122,123,127]. Furthermore, 52% of patients with *BRCA1/2* mutations had a confirmed PSA response, where there was a 50% decrease in the PSA level [127]. Based on the positive preliminary findings, the FDA approved rucaparib for *BRCA1/2* mCRPC patients progressing after first line treatment in May 2020 [65].

GALAHAD is an ongoing phase II trial of 165 patients with mCRPC, of which 81 had biallelic alterations (46 BRCA and 35 non-BRCA) and 47% of patients had visceral metastases [124]. Patients included in this trial had mCRPC disease progression despite first line treatment of taxane-based chemotherapy and AR signaling inhibitor and were treated with niraparib 300 mg once a day. Results showed that patients with *BRCA1/2* mutations had the highest ORR (41%) compared to patients without *BRCA* mutations (9%). The composite response rate (CRR) was defined as ORR, conversion of CTC to <5/7.5 mL blood or ≥50% decline in PSA. The CRR was 63% in BRCA patients and 17% in those without *BRCA1/2* mutations. Therefore, niraparib has shown potential for response in mCRPC, especially in carriers of biallelic *BRCA1/2* mutations. It is important to note that the GALAHAD study confirmed patient eligibility with biallelic mutations, whilst TRITON-1 and PROFOUND trials evaluated mono- and biallelic mutations in patients’ tumor tissue or plasma and tumor tissue, respectively [65]. It is unknown if the origin and type of *BRCA1/2* mutation (monoallelic vs biallelic, somatic vs germline) could affect therapeutic response to PARP inhibitors. 

PARP inhibitors are the first registered treatment for mCRPC based on the synthetic lethality mechanism [54]. The main reservations concerning PARP inhibitors studies includes proper genetic analysis with most genetic testing performed in less optimal sources such as saliva and blood samples, primary tumor tissue and circulating DNA; whilst most optimal source would be from freshly collected tumor biopsy, despite the reliance of HRR status on the stratification to treatment [54]. The PROFOUND study suggested that olaparib may be ineffective in managing tumors with ATM mutation; consistent with the phase 2 trials TOPARP-B and TRITON2 studies showing a general lack of response to rucaparib and olaparib to mCRPC with non-*BRCA* mutations [54,120,121,122]. The use of PARP inhibitors may be employed beyond *BRCA1/2* mutation but also on tumors described as ‘BRCAness’, whereby they share the *BRCA1/2* phenotype rather than genotype [128,129]. Considering that prostate cancer is determined by a combination of altered signaling pathways, the studies implicate the importance of future trials investigating biomarkers beyond BRCA to predict the benefit of PARP [130,131].

### 5.3. PARP Inhibitors and AR Signalling Inhibitors

The AR is a transcription factor that influences the growth and progression of prostate cancer [132]. ADT targets the AR signalling pathways and is an efficient first-line treatment for advancing prostate cancer. The AR signalling pathway has also been implicated in mCRPC [10]. Robinson et al. identified AR pathway aberrations in 71.3% of cases (both amplification and mutations) through direct mutations of AR along with other genes including *NCOR1*, *NCOR2*, and *FOXA1* [63]. Understanding these mutations is vital to improve treatment options for metastatic prostate cancer. AR signalling inhibitors down-regulate expression of DDR gene expression and increase DNA damage, therefore increasing the sensitivity of prostate cancer to PARP inhibitors [133,134]. Asim et al. showed that ADT impaired HR and blocked AR signalling, which activated PARP signalling—however, with PARP inhibition (use of olaparib), synthetic lethality was induced as there was decreased tumor volume [132]. Li et al. also showed there was a superior effect with DNA damage-induced apoptosis when enzalutamide was followed by olaparib compared to monotherapy with olaparib or enzalutamide [134]. ADT therefore creates a state of ‘BRCAness’ when both PARP and AR signalling are inhibited and so PARP inhibitors may have a role beyond DDR mutated prostate cancers [41].

### 5.4. PARP Inhibitors and Immune Checkpoint Inhibitors 

There is also growing evidence into the synergy between PARP inhibitors and immune checkpoint inhibitors [41,135]. The ligand programed death ligand-1 (PD-L1) maintains an immunosuppressive tumor microenvironment and is activated in prostate cancer; inhibition of PD-L1 may allow effective T-cell responses against tumor cells [136,137,138]. Furthermore, PARP inhibition increases PD-L1 expression in cells with reduced BRCA2 expression [139]. PD1 blockers, such as pembrolizumab and nivolumab, and PD-L1 blockers such as durvalumab, are being combined with PARP inhibitors in clinical trials [41]. Karzai et al. showed efficacy of combining olaparib with durvalumab in mCRPC with 53% of patients having an over 50% decline in PSA, and median radiographic PFS in patients with DDR defects being 16.1 months compared to 4.8 months in patients without [140]. There are ongoing trials investigating use of PARP inhibitors with immune checkpoint inhibitors in mCRPC [141]. 

In summary, PARP inhibitors have a growing role in treatment of prostate cancers with underlying mutations in the HRR pathway—specifically *BRCA1/2* [142]. Use of PARP inhibitors has shown particularly favorable outcomes in *BRCA1/2* mutations with increased survival outcomes [142]. Olaparib has a good curative effect and is used in prostate cancer with *BRCA* mutations that progressed following novel hormonal agents, e.g., enzalutamide or abiraterone; Rucaparib is used in combination with AR guided therapy and paclitaxel-based chemotherapy in mCRPC with harmful *BRCA* mutations [143,144]. The fact that a fraction of biomarker-negative patients respond to PARP inhibitors is indicative of undetected DDR mutations that may exist in genomic regions less effectively covered by NGS. Olaparib has been approved by the FDA for mCRPC with genomic alterations in 14 different DNA repair genes, whilst only for BRCA-mutant cancers by the European Medicines Agency (EMA). Rucaparib has been approved by the FDA for the treatment of mCRPC patients with somatic and/or germline DDR alterations who have progressed through enzalutamide or abiraterone treatment. With ongoing trials, niraparib and talazoparib are awaiting FDA approval in prostate cancer treatment. However, further trials are studying PARP inhibitor use in a wider range of DDR genes, though the results have been variable and inconsistent as not all DDR mutations produce an equal response to PARP inhibitors [144]. This may be due to differences each gene mutation may have on DNA repair and differences in individual PARP inhibitors [142]. 

Overall PARP inhibitors are a revolutionary treatment in the management of mCRPC in the era of targeted therapies; with the future view of enabling personalized therapy with effective treatment results by determining prognostic and predictive factors as well as individual gene contributions [54,108]. Several trials are in place to assess PARP inhibitor efficiency at different tumor stages as a monotherapy or polytherapy regime, and these will change the future of use of PARP inhibitors in years to come [54]. 

## 6. Safety and Toxicity Profile of PARP Inhibitors

The safety profile of PARP inhibitors in patients with mCRPC is similar to patients with other solid tumor types and fatigue, gastrointestinal adverse effects, and myelosuppression are among the most common adverse events reported [145,146]. In comparison to chemotherapy, the toxicity of PARP inhibitors is low; however, more studies about the long-term safety of PARP inhibitors in prostate cancer populations are needed. According to the PROFOUND trial, the most common adverse events were haematological (anaemia, 46%), gastrointestinal (nausea, 41%, loss of appetite, 30%) and fatigue or asthenia (41%) [121]. Dose reduction was required in 22% of the patients due to adverse events. The GALAHAD and TRITON2 studies also established that the most common adverse event is anaemia at 17.9–25% [122,123,124]. Another study reported anaemia was present in 50% of patients treated with PARP inhibitors [147]. A meta-analysis of 14 trials showed that PARP inhibitors in combined therapy may be associated with myelodysplastic syndrome, but the incidence of this complication is low [147]. Furthermore, elevations in alanine transaminase (ALT), aspartate transaminase (AST), and creatinine were commonly reported; however, there was no associated hepatic or renal toxicity. Elevated creatinine is suggested to be caused by inhibition of renal transporters (e.g., MATE-1, MATE2-K, OCT2) rather than directly impacting renal function, but creatinine levels typically stabilise by the third week [121,148]. Vomiting, diarrhoea, and dizziness are also reported in around 20–25% of patients; however, the severity is mild [122]. 

## 7. Resistance Mechanisms to PARP Inhibitors

**Figure 2:**
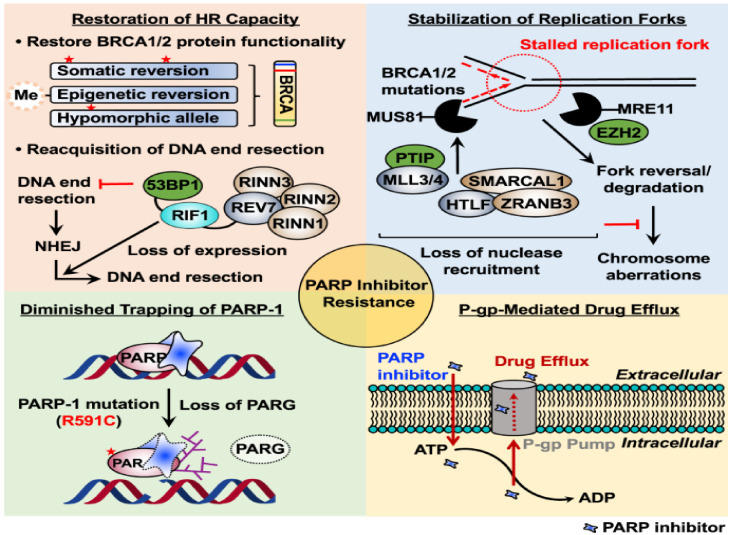
Mechanisms of resistance to PARP inhibitors. Cancer cells develop different resistance mechanisms that pose a significant obstacle to the prolonged use of PARPi. Several proposed molecular mechanisms of PARPi resistance, including restoration of HR capacity, stabilization of replication forks, diminished trapping of PARP-1, and P-gp-mediated drug efflux, are highlighted in four sections.

There are a few mechanisms that cause PARP inhibitors’ resistance. Firstly, tumor cells can perform mutational reversion of *BRCA1/2*, which causes restoration of the HR DNA repair pathway [149]. Secondly, the stabilization of stalled forks in the cells also leads to resistance; this is achieved through the loss of fork degradation pathways [124]. A third mechanism is through the mutation of PARP1 in tumor cells preventing trapping and cytotoxicity to these cells [124]. Lastly, tumor cells can upregulate the permeability glycoprotein (PgP) efflux pumps, causing reduction of PARP inhibitors concentration intracellularly and decreased efficacy [124]. The summary of the mechanisms of resistance to PARP inhibitors is presented in Figure 2. 

### 7.1. Reversion Mutation

The most common HR-disrupting mutations in tumor cells involving *BRCA1* and *BRCA2* are single-nucleotide mutations, short insertions or deletions that lead to frame-shifts [124]. Secondary mutations would enable correction and potentially restore protein activity. Studies on cell line models that resulted in PARP inhibitors’- resistant clones have shown high occurrence rates of such mutations [150]. These reversion mutations arise from the repair of DSBs through alternative error-prone mechanisms in the original HR-deficient cells [151]. The effects of reversion mutation are not exclusive to PARP inhibitors resistance but also as resistance mechanisms for other DNA-damaging drugs [152].

### 7.2. Increase of Drug Efflux

Most tumors that displayed resistance to PARP inhibitors showed overexpression of drug-efflux transporter genes (Abcb1a and Abcb1b encoding for MDR1/P-gp, and Abcg2) [153]. These include studies that have observed PARP inhibitors’ resistance in human ovarian cancer cell line and mouse mammary tumors [154,155].

### 7.3. Decreased PARP Trapping through Disruption of PARP1 and PARG Proteins

PARP proteins act as a catalyst to modify proteins by covalent addition of PAR chains—this process is known as PARylation [124]. When DNA is damaged, PARP1 is the main protein involved in most cellular PARylation [156]. A good predictor for cytotoxicity is the capacity of the different inhibitors to trap PARP proteins on damaged chromatin. PARP1 is the most abundant PARP protein in cells, and it accounts for more than 90% of cellular PARylation [157]. As a result, PARP1 mutations that reduce the trapping of the protein on DNA induce PARP inhibitors’ resistance even in HR-deficient cells [126]. Additionally, PAR glycohydrolase (PARG) reverses PARylation and is responsible for the degradation of PAR chains [158]. Therefore, PARG works in the same way as PARP inhibitors by preventing PAR accumulation. A study performed on genetic screening of BRCA2-deficient mice cell lines has identified loss of PARG as another cause for PARP inhibitors’ resistance [133].

### 7.4. Stabilization of Stalled Forks

During replication stress, cells undergo arrest to allow time for repair and re-entry into the cell cycle [159]. BRCA1 and BRCA2 are responsible for the protection of stalled replication forks [134,160]. In the absence of BRCA1/2, nucleases such as MRE11 and MUS81 attack stalled replication forks, leading to fork collapse and chromosomal abnormality. PARP inhibitors’ sensitivity is affected by factors involved in recruiting these nucleases to the stalled replication fork, such as EZH2 and PTIP [161]. The cytotoxicity of PARP inhibitors’ treatment results from fork degradation of deprotected replication forks in *BRCA1* or *BRCA2*-mutated cells. There are at least three mechanisms that mediate fork degradation, and therefore loss of these mechanisms leads to protection of the fork and thereby PARP inhibitors’ resistance. The degradation is affected by factors involved directly or indirectly in fork remodelling and replication stress, such as chromatin-remodelers SMARCAL1, ZRANB3, HLTF, PTIP and SLFN11. The depletion of these factors will consequently lead to PARP inhibitors’ resistance [162].

## 8. Conclusions

Prostate cancer is the second most common cancer in men worldwide and is a complex heterogeneous disease with high inheritability. Germline and somatic mutations have been found, especially in the *BRCA* genes and subsequently national guidelines such as the NCCN are recommending germline and somatic testing. This has changed the molecular classification of prostate cancer and therefore aids therapeutic options in these patients along with implications for screening of their relatives. PARP inhibitors have been a significant development in prostate cancer. They were initially thought to be relevant for DDR mutations, but their use is expanding with combined use with AR signalling inhibitors and immune checkpoint inhibitors. Further clinical trials are ongoing to assess their efficacy in this regard with potential for an increased patient population to be targeted; this would be significant in reducing the morbidity and mortality that is prevalent with prostate cancer. 

## Figures and Tables

**Table 1:** Comparison of the percentage of germline DNA damage response mutations in prostate cancer.

Gene	DDR Pathway	Percentage (%) of Germline Mutations in Tested Prostate Cancer Patients	
		Nicolosi et al. [75]	Pritchard et al. [61]
*ATM*	Homologous Recombination	2.03	1.59
*ATR*	Homologous Recombination	Not tested	0.29
*BRCA1*	Homologous Recombination	1.25	0.87
*BRCA2*		4.74	5.35
*BARD1*	Homologous Recombination	0.00	0.00
*BRIP1*	Homologous Recombination	0.28	0.18
*CDKN2a*	p16/cyclin-dependent kinase/retinoblastoma gene pathway	0.13	Not tested
*CHEK2*	Homologous Recombination	2.88	1.87
*FAM175A*	Homologous Recombination	Not tested	0.18
*GEN1*	Homologous Recombination	Not tested	0.46
*MLH1*	Mismatch Repair	0.06	0.00
*MSH2*		0.69	0.14
*MSH6*		0.45	0.14
*NBN*	Homologous Recombination	0.32	0.29
*PALB2*	Homologous Recombination	0.56	0.43
*PMS2*	Mismatch Repair	0.54	0.29
*RAD51C*	Homologous Recombination	0.21	0.14
*RAD51D*	Homologous Recombination	0.15	0.43

**Table 2:** Clinical trials of poly(ADP-ribose) polymerase inhibitors.

Clinical Trial	Phase	Study PARPi	Strategy	Primary Endpoint
TOPARP-A [67]	II	Olaparib	Olaparib in mCRPC	RR, PSA, CTC
TOPARP-B [120]	II	Olaparib	Olaparib in mCRPC with gene analysis	RR, PSA, CTC
PROFOUND [121]	III	Olaparib	Olaparib vs ARSi with gene analysis	rPFS
TRITON2 [122,123]	II	Rucaparib	Rucaparib in mCRPC	ORR
GALAHAD [124]	II	Niraparib	Niraparib in mCRPC	ORR
TALAPRO-1 [119]	II	Talazoparib	Talazoparib in mCRPC	ORR

Abbreviations: TOPARP: trial of PARP Inhibitor Prostate Cancer; mCRPC: metastatic castration resistant prostate cancer; ARSi: androgen receptor signaling inhibitor; RR: response rate; PSA: Prostate Specific Antigen; CTC: circulating tumor cells; rPFS: radiographic progression-free survival; ORR: objective response rate.
